# Clinical and Epidemiologic Characteristics of 3 Early Cases of Influenza A Pandemic (H1N1) 2009 Virus Infection, People’s Republic of China, 2009

**DOI:** 10.3201/eid1509.090794

**Published:** 2009-09

**Authors:** Cao Bin, Li Xingwang, Shu Yuelong, Jiang Nan, Chen Shijun, Xu Xiayuan, Wang Chen, for the National Influenza A Pandemic (H1N1) 2009 Clinical Investigation Group

**Affiliations:** Capital Medical University, Beijing, People's Republic of China (C. Bin, W. Chen); Beijing Ditan Hospital, Beijing (L. Xingwang); Chinese Center for Disease Control and Prevention, Beijing (S. Yuelong); Sichuan Province People’s Hospital, Chengdu, People's Republic of China (J. Nan); Jinan Infectious Diseases Hospital, Jinan, People's Republic of China (C. Shijun); Peking University, Beijing (X. Xiaoyuan)

**Keywords:** influenza, influenza A pandemic (H1N1) 2009 virus, viruses, China, research, expedited, podcast

## Abstract

On May 7, 2009, a national network was organized in the People’s Republic of China for the surveillance, reporting, diagnosis, and treatment of influenza A pandemic (H1N1) 2009 virus infection (pandemic [H1N1] 2009). Persons with suspected cases are required to report to the Chinese Center for Disease Control and Prevention and the Ministry of Health within 24 hours; the patient’s close contacts are then traced and placed in quarantine for 7 days. We report 3 confirmed early cases of pandemic (H1N1) 2009. Two cases were imported from United States; the other was imported from Canada. The patients exhibited fever and signs and other symptoms that were indistinguishable from those of seasonal influenza. Serial virologic monitoring of pharyngeal swabs showed that they were negative for pandemic (H1N1) 2009 virus by real-time reverse transcription–PCR 4–6 days after onset of illness. One close contact whose sample tested positive for pandemic (H1N1) 2009 virus had no symptoms during quarantine. A national network is essential for controlling pandemic (H1N1) 2009.

## Abstract 2

A national network is essential for controlling this infection.

In early April 2009, human infections caused by an influenza virus designated as influenza A pandemic (H1N1) 2009 virus (pandemic [H1N1] 2009) were identified in United States (*1*,*2*) and Mexico (*3*) and spread rapidly to other regions of the world (*4*,*5*). Pandemic (H1N1) 2009 virus has been reported to be a triple reassortant influenza virus (containing genes from human, porcine, and avian influenza viruses) that has circulated among swine in the United States since 1999 (*6*–*8*). Sporadic human infections by influenza virus of swine origin had been described, mostly in young persons in contact with pigs (*9*). The current outbreak indicates that the new pandemic (H1N1) 2009 virus can be transmitted from human to human. As of June 17, 2009, a total of 88 countries officially reported a cumulative total of 39,620 laboratory-confirmed pandemic (H1N1) 2009 cases; these cases occurred in Mexico (6,241 cases [including 108 deaths]), the United States (17,855 [44 deaths]), Canada (4,049 [7 deaths]), Chile (2,335 [2 deaths]), Argentina (733 [1 death]), the United Kingdom (1,461 [1 death]), Costa Rica (149 [1 death]), Australia (2,112), Japan (666), the People’s Republic of China (382), Germany (195), France (118), and other countries (*10*). In this report, we describe the clinical and epidemiologic characteristics of 3 early confirmed cases of pandemic (H1N1) 2009 in China.

## Methods

### Surveillance, Reporting, and Data Collection

On May 7, 2009, a national network for monitoring pandemic (H1N1) 2009 was organized in China. The partners included the Ministry of Health (MOH), the Chinese Center for Disease Control and Prevention (Chinese CDC), and all community hospitals and teaching hospitals. A guideline for the surveillance, reporting, diagnosis, and treatment of pandemic (H1N1) 2009 was published on May 9, 2009. Any ill persons with a body temperature >37.5°C were asked to visit the fever clinics in local general hospitals.

A confirmed case of human infection with pandemic (H1N1) 2009 virus was defined as laboratory confirmation of infection from a human sample at the Chinese CDC or the Chinese Academy of Medical Sciences (CAMS) (see the Laboratory Confirmation section below). A suspected case was defined as 1) an influenza-like illness (ILI; fever >37.5°C with at least 1 symptom or sign, including sore throat, cough, rhinorrhea, nasal congestion) in a person who has traveled to a country where >1 confirmed pandemic (H1N1) 2009 cases had been found in the past 7 days, or 2) clinical symptoms or signs of ILI in a person with an epidemiologic link to a patient found to have confirmed or suspected pandemic (H1N1) 2009 in the previous 7 days.

Close contacts were defined as persons who had lived with a person with probable or confirmed pandemic (H1N1) 2009 or who had had direct contact with the respiratory secretions or body fluids of such persons. All close contacts were quarantined for medical observation for 7 days; during this time, pharyngeal swabs were collected for identification of pandemic (H1N1) 2009 virus (once if results were negative; serial repeat tests if results were positive).

All suspected cases were required to be reported to the Chinese CDC and to the MOH within 24 hours of diagnosis. Pharyngeal swabs were forwarded to a local branch of the Chinese CDC for detection of influenza A pandemic (H1N1) 2009 virus by real-time reverse transcription–PCR (RT-PCR). All patients with a positive RT-PCR result for pandemic (H1N1) 2009 virus were admitted to the local infectious disease hospital where the patient was placed in isolation. Additional specimens were sent to the State Reference Influenza Laboratories at the Chinese CDC or CAMS for further characterization and nucleic acid sequencing.

A standardized surveillance reporting form was used to collect clinical, epidemiologic, or demographic data. We included information about the patient’s demographic characteristics, underlying medical conditions, status with respect to seasonal influenza vaccination, exposures to swine and other animals, travel to a country with confirmed pandemic (H1N1) 2009 infection, clinical signs and symptoms, chest radiograph results, laboratory findings, results of diagnostic testing for influenza, antiviral treatment, clinical complications, and clinical outcome.

### Laboratory Confirmation

Pharyngeal swabs were collected from all patients and their close contacts, which were submitted to local branch of the Chinese CDC and reference laboratories in the Chinese CDC or CAMS for investigation. We used the protocol of the US Centers for Disease Control and Prevention of real-time RT-PCR for pandemic (H1N1) 2009 as recommended by the World Health Organization (*11*). The PCR products were sequenced for further confirmation by standard high-throughput sequencing system with the use of BigDye Terminator, version 3.1 (Applied Biosystems, Foster City, CA, USA) with 1 mm^3^ of double-stranded template.

## Results

The demographic and epidemiologic characteristics of the 3 patients with confirmed pandemic (H1N1) 2009, including the estimated disease incubation period and their travel histories, are listed in Table 1. Their clinical characteristics are shown in Table 2. All 3 patients were Chinese students who had been studying abroad (2 in United States and 1 in Canada).

**Table 1:** Demographic and epidemiologic characteristics of 3 patients infected with influenza A pandemic (H1N1) 2009 virus, People’s Republic of China, 2009

Characteristic	Patient 1	Patient 2	Patient 3
Age, y	30	19	18
Sex	M	M	F
City of origin	St. Louis, MO, USA	Winnipeg, Manitoba, Canada	New York, NY, USA
Date of illness onset	May 9	May 10	May 13
Site of illness onset	Airplane	Hotel	Home
City of illness onset	Beijing	Beijing	Beijing
City of virus isolation	Chengdu	Jinan	Beijing

**Table 2:** Clinical characteristics of 3 patients infected with influenza A pandemic (H1N1) 2009 virus, People’s Republic of China, 2009*

Characteristic	Patient 1	Patient 2	Patient 3
Chronic illness	No	No	No
Influenza vaccine in last flu season	No	No	No
Highest temperature, °C	38.8	39	39.4
Sore throat	Yes	Yes	Yes
Cough	Yes	No	Yes
Rhinorrhea	Yes	No	Yes
Nasal congestion	Yes	No	No
Headache	Yes	Yes	Yes
Diarrhea	No	No	No
Other symptoms	No	Decreased appetite	Chest pain
Leukocyte count, per mm^3^	7,900	4,900	5,300
Neutrophil count, per mm^3^	5,480	2,810	4,000
Lymphocyte count, per mm^3^	1,540	1,580	1,000
Platelet count, per mm^3^	166,000	208,000	254,000
C-reactive protein, mg/L	39	Not done	0.3
Findings on chest radiograph	Normal	Normal	Normal
Oseltamivir treatment*	Yes	Yes	Yes
Duration of fever, d	2	3	3
Length of stay in hospital, d†	8	8	8
Outcome	Recovered	Recovered	Recovered

*75 mg 2x/d for 5 d. †During their stay in hospital, no patient needed oxygen or a ventilator. They were kept in hospitals for isolation and close observation because pandemic (H1N1) 2009 was an infectious disease new to physicians in China.

Patient 1 left St. Louis, Missouri, USA, on May 7, 2009 (US date), and flew to St. Paul, Minnesota, USA, where he transferred to a flight to Tokyo, Japan, then to Beijing, China, arriving on May 8 (Beijing date). The patient did not experience illness, and fever did not develop during the flight from St. Louis to Beijing. At the Beijing airport, his temperature was normal. The patient stayed in Beijing for 1 night and boarded a flight to Chengdu on May 9. He did not report feeling ill before the flight. On the flight from Beijing to Chengdu, fever developed. Upon landing, the patient went to a hospital for medical attention for his illness. The case was reported to the Chinese CDC, and specimens were collected on May 9. A test result for pandemic (H1N1) 2009 virus was positive on May 10 by real-time RT-PCR. All 144 close contacts (including his father, girlfriend, taxi driver, and all passengers on the flight from Chendu to Beijing) were quarantined in hotels for medical observation for 7 days.

Patient 2 left Winnipeg, Manitoba, Canada, on May 7 (Canada date) and arrived in Beijing on May 8 (Beijing date). He did not feel ill while traveling from Winnipeg to Beijing. He stayed for 2 days in Beijing, where a fever developed, but he did not seek medical attention. The patient subsequently took a train from Beijing to Jinan on May 11 and, on arrival, went to the local infectious diseases hospital. There was no thermal scanner for fever at the train station in Beijing. The case was reported to the Chinese CDC, and specimens were collected on May 11. The pandemic (H1N1) 2009 virus test result was positive on May 12 by real-time RT-PCR. All 40 close contacts sitting in the same car of the train were quarantined for medical observation for 7 days.

Patient 3 left New York, New York, USA, on May 10 (US date) and arrived at home in Beijing on May 11 (Beijing date). She was well when traveling from New York to Beijing. Her temperature was normal at border health/temperature monitoring. She stayed home with her mother and did not meet other persons and visit other places. A fever developed on May 13 (Beijing date), and she sought medical attention on the evening of May 14. The case was reported to Chinese CDC, and specimens were collected on May 15. The test result for pandemic (H1N1) 2009 virus was positive on May 15 by real-time RT-PCR. Her mother and a taxi driver were the only close contacts.

All 3 patients were positive for pandemic (H1N1) 2009 virus by real-time RT-PCR, and further confirmed PCR products sequencing the partial hemagglutinin sequence from all 3 patients were 99.5–100% homologous with A/California/4/2009 but only 79.4%–79.6% homologous with seasonal influenza virus (H1N1) (A/Brisbane/59/2007) (Table 3). The dynamic virologic monitoring of pharyngeal swab samples from the 3 case-patients showed that real-time RT-PCR results for influenza A pandemic (H1N1) 2009 virus were negative on day 5, day 7, and day 6, respectively (Table 3).

**Table 3:** Dynamic virologic monitoring of influenza A pandemic (H1N1) 2009 virus by real-time RT-PCR of 3 patients and the mother of patient 3, People’s Republic of China, 2009*

Day no.	Patient 1	Patient 2	Patient 3	Mother of patient 3
1	+ (May 9)	ND (May 10)	ND (May 13)	ND (May 13)
2	+	+	ND	ND
3	+	+	–	ND
4	+	+	+	ND (patient 3 isolated)
5	–	+	+	+
6	–	+	–	+
7	–	–	–	–
8	Discharged	–	–	–
9		Discharged	–	–
10			Discharged	–

*Day 1 of patients 1, 2, and 3 indicates the day of onset of fever. Day 1 of the mother of patient 3 indicates the day when fever developed in patient 3. Gray shading indicates days of oseltamivir treatment. RT-PCR, reverse transcription–PCR; ND: not done; +, positive for influenza A pandemic (H1N1) 2009 virus; –, negative for influenza A pandemic (H1N1) 2009 virus.

Only 1 sample from a total of 186 close contacts tested positive for pandemic (H1N1) 2009 virus by real-time RT-PCR. This sample came from the 48-year-old mother of patient 3, who had lived with patient 3 for 2 days before her illness and had taken care of her for 2 days after her illness began. On the fifth day (May 16) after exposure to patient 3, the woman’s sample became positive (Table 3). As with all the other close contacts, this woman had no fever or ILI symptoms.

## Discussion

The 3 cases were mild, and the patients were young, which is consistent with the profile of other pandemic (H1N1) 2009 infections reported around the world (*6*). The most frequent signs and symptoms in the patients were fever and other manifestations that were nonspecific for pandemic (H1N1) 2009 and indistinguishable from those of seasonal influenza. None of them had evidence of severe lower respiratory tract illness or unusual symptoms of influenza, such as diarrhea. All patients recovered quickly, with a median duration of fever of 3 days. All 3 patients had complete blood counts performed during the course of their disease, but none had leukopenia (leukocyte count <4,000/mm^3^), or lymphopenia (total lymphocyte count <800/mm^3^), or thrombocytopenia (total platelet count <100,000/mm^3^).

The transmissibility of influenza A pandemic (H1N1) 2009 virus is uncertain. One study assumed that its transmissibility (*R*_0_) is substantially higher than that of seasonal influenza and comparable with the viruses in previous influenza pandemics (*12*). Our study demonstrates, on the basis of PCR testing, that the average time of pandemic (H1N1) 2009 virus shedding is 4–6 days. Positive RT-PCR results do not necessarily confirm virus shedding, but the PCR-based method is more sensitive than culture methods for detecting virus shedding (*13*). Our study also shows that the risk for person-to-person transmission is greatest in households. Among 186 close contacts, only the mother of patient 3, who lived with her, had a positive result for pandemic (H1N1) 2009 virus by real-time RT-PCR, although she had no symptoms of influenza. All other close contacts, including taxi drivers, passengers in an airplane or the same car of a train, were negative for pandemic (H1N1) 2009 virus at screening, and illness did not develop subsequently while they were being observed. Asymptomatic infections appear to be possible among household members, as demonstrated by case-patient 3 and her mother.

Our study has several limitations. First, numbers in this series were low. Second, the exact date of exposure to a known infectious source was difficult to trace. Third, pharyngeal swab samples may have a lower sensitivity (than nasopharyngeal), and thus some false-negative results might have occurred, which could lead to underestimation of viral shedding.

A national network for the surveillance and control of pandemic (H1N1) 2009 was quickly organized in China, and the response was quick and thorough. The Chinese government is moving swiftly to contain the new influenza, drawing on lessons from the severe acute respiratory syndrome and bird influenza outbreaks in recent years. In all airports, thermal scanners have been installed, and all asymptomatic contacts of case-patients were quarantined for 7 days as part of the early response. As the number of imported cases has increased, the quarantine policy is changing. During the past 4 weeks, only symptomatic travelers at ports of entry and passengers sitting within a short distance (<2 m) to a person with a suspected or confirmed case were quarantined. As local transmission has been documented, more efforts have been paid to communities. Since June 22, hand temperature monitors were used in all schools in Beijing. All students with body temperatures >37.2°C were asked to stay at home until their temperature returned to normal. Further evaluation is needed to continue to clarify the nature of pandemic (H1N1) 2009, including its clinical features, severity, incubation period, and transmission patterns (*14*).
